# Small Pox

**Published:** 1834

**Authors:** 


### VII. Small Pox.

Since the cessation of Epidemic Cholera in autumn, the small pox has been present, though not prevalent in our city. The cases have been scattering, and although several persons have died, there has been no alarm. Had the city been thus invaded before the discovery of vaccination, the mortality, in proportion to its population, would have been very great. Immediately on the appearance of the epidemic, the Cincinnati Medical Society, ever solicitous for the public health, appointed a committee of two members—Drs. L. C. Rives and Wm. Judkins—as dispensers of Vaccine Infection; and our brethren in the country can at all times obtain supplies of fresh and genuine infection, gratuitously, by writing (post paid) to either of these gentlemen.

